# Determinants of Poor Glycemic Control in Type 2 Diabetes Patients With Coronary Artery Disease: A Case‐Control Study

**DOI:** 10.1002/hsr2.71872

**Published:** 2026-02-23

**Authors:** Habib Haybar, Maryam Memar, Seyed Masoud Seyedian, Shirin Azizidoost, Shooka Mohammadi

**Affiliations:** ^1^ Department of Cardiology, School of Medicine Ahvaz Jundishapur University of Medical Sciences Ahvaz Iran; ^2^ Atherosclerosis Research Center Ahvaz Jundishapur University of Medical Sciences Ahvaz Iran

**Keywords:** coronary artery disease, diabetes mellitus, glycemic control, HbA1c, T2DM

## Abstract

**Background and Aim:**

Poor glycemic control increases the risk of cardiovascular complications in patients with type 2 diabetes mellitus (T2DM) and coronary artery disease (CAD). The current study identified determinants of poor glycemic control in Iranian T2DM patients with CAD.

**Methods:**

This case–control study included T2DM patients with CAD who were admitted to Golestan Hospital in Ahvaz (Iran) during 2 years. Demographic, clinical, and biochemical data were collected. According to their glycemic control status, patients were divided into two groups: uncontrolled glycemia (Group 1, *n* = 350; hemoglobin A1c [HbA1c] > 7%) and controlled glycemia (Group 2, *n* = 350; HbA1c ≤ 7%).

**Results:**

The study assessed 700 T2DM patients with CAD (458 males and 242 females). Their average age was 60.51 ± 9.76 years. Compared to patients with optimal glycemic control, those with uncontrolled glycemia were younger, had higher rates of dyslipidemia, previous myocardial infarction, smoking, chronic kidney disease, hypertension, peripheral artery disease, diabetes for a longer period, higher body mass index (BMI), lower ventricular ejection fraction, greater levels of total cholesterol, HbA1c, fasting blood glucose, triglycerides, triglyceride‐glucose (TyG) index, and low‐density lipoprotein cholesterol (*p* < 0.05). In addition, there were substantial differences in the prevalence of New York Heart Association (NYHA) functional class, angiographic findings (including severe calcification, type B2/C lesions, chronic total occlusion lesions, thrombotic lesions, and ostial lesions), as well as the use of oral glucose‐lowering medications and insulin between the two groups (*p* < 0.05). Furthermore, 42.6% of patients exhibited triple vessel disease, and 60% of them had HbA1c level > 7%. Diabetes duration, BMI, and TyG index were significant independent predictors of glycemic control.

**Conclusion:**

This study revealed that CAD patients with good glycemic control had better clinical and cardiovascular status. HbA1c may serve as a valuable parameter for risk stratification in these patients.

## 1 Introduction

Cardiovascular disease (CVD) is the primary cause of mortality among patients with type 2 diabetes mellitus (T2DM) [1]. Diabetes has a strong correlation with CVDs, particularly coronary artery disease (CAD) [2]. T2DM is a significant risk factor for CAD that accelerates the progression of atherosclerotic cardiovascular disease (ASCVD) [3, 4]. Patients with T2DM are more susceptible to developing CAD, which results in higher morbidity and mortality rates [5, 6, 7]. They have significantly higher risks of experiencing adverse cardiovascular events in the future than those without diabetes who have CAD [8]. It has been indicated that patients with T2DM and CAD exhibit higher levels of stenosis in the coronary arteries, emphasizing the need for regular screening to detect silent CAD and prevent adverse outcomes [7, 9, 10].

The prevalence of T2DM and disability‐adjusted life years (DALYs) is increasing worldwide, especially in areas with low to middle sociodemographic indices [11]. Iran recorded 5,035,012 cases of T2DM in 2019, which is a notable 417% increase in the number of cases compared to 1990 [12]. Complications such as CVDs (33%) and nephropathy (43%) are highly prevalent among Iranian T2DM patients [13].

Chronic hyperglycemia is significantly related to elevated risks of cardiovascular complications [14]. Consequently, achieving lower glycemic levels may improve clinical outcomes for patients with diabetes [15]. The management of glucose control in diabetic patients with CAD continues to be a topic of debate [16]. A meta‐analysis related to patients with T2DM who exhibited at least one CVD risk factor found that intensive glycemic control led to a 9% reduction in the occurrence of major adverse cardiovascular events [17]. This risk reduction was primarily linked to decreased incidences of myocardial infarction (MI) [17]. Moreover, achieving optimal glycemic control may slow the development of coronary artery atherosclerosis and calcification in asymptomatic T2DM patients without a prior history of stroke or CAD [18].

Understanding the complex association between T2DM and CAD [19, 20] is crucial to developing effective management and prevention strategies for patients with T2DM who are at risk of CAD [21] to reduce rehospitalization rates and improve healthcare quality [22, 23]. Patients with CAD and T2DM face a complex interplay of cardiovascular risk factors [24]. Managing these factors effectively can reduce the risk of complications [25, 26]. There are challenges in managing key risk factors in these patients, with a significant number of cases showing ineffective glycemic and dyslipidemia control or hypertension (HTN) management [27].

Glycated hemoglobin or hemoglobin A1c (HbA1c), an important indicator of glycemic control, is considered a more reliable predictor of CAD than fasting plasma glucose or 2‐h plasma glucose measurements in patients with diabetes [28, 29, 30]. HbA1c reflects the average blood glucose levels in patients with T2DM over 3 months. Achieving an HbA1c target of less than 7% could reduce the risk of vascular complications related to diabetes [31]. A reduced incidence of macrovascular and microvascular complications in patients with T2DM was associated with optimal glycemic control [32]. Very few studies have evaluated glycemic control and associated factors among T2DM patients with CAD in Iran [33, 34]. This study aimed to assess the determinants of poor glycemic control in Iranian T2DM patients with CAD.

## 2 Materials and Methods

This case‐control study evaluated glycemic control and associated factors in T2DM patients with CAD admitted to the cardiology clinic of Golestan Hospital in Ahvaz (Iran) from January 2023 to January 2025. Before the commencement of the study, informed consent was obtained from all participants.

Diabetes was recorded if the patients received glucose‐lowering therapy, had a history of diabetes, exhibited a fasting blood glucose (FBG) level of ≥ 7.0 mmol/L or ≥ 126 mg/dL, had a HbA1c level of ≥ 6.5%, or demonstrated a 2‐h plasma glucose level of ≥ 11.1 mmol/L during an oral glucose tolerance test [35]. CAD was confirmed through angiography and characterized by the identification of coronary stenosis of 50% or greater in at least one major arterial segment. This evaluation was conducted by two qualified cardiologists based on the findings from the coronary angiography. Patients were categorized into two groups based on their glycemic control status. Group 1 (cases) consisted of patients with poor glycemic control, defined as those with HbA1c level > 7%. In contrast, Group 2 (control) comprised patients with optimal glycemic control, characterized by HbA1c level ≤ 7%.

The study focused on T2DM patients with CAD aged 18 to 80 years. A convenience sampling method was applied to identify participants who met the specified inclusion criteria. The primary exclusion criteria included the lack of comprehensive laboratory data (specifically fasting triglycerides (TG) and FBG), age restrictions (individuals younger than 18 years or older than 80 years), hepatic, lung, or renal impairment, decompensated heart failure (HF), ejection fraction (EF) below 30%, systemic inflammatory conditions, the presence of malignant neoplasms, and acute infectious diseases. The diagnoses of diseases listed as exclusion criteria were based exclusively on existing clinical diagnoses documented in the medical records of patients before enrollment. The research team did not establish any additional diagnostic criteria and relied solely on the information provided in the medical records. All diagnoses had been made by physicians using appropriate laboratory tests as part of routine medical care and were documented in official medical records, which were reviewed during the screening process.

### 2.1 Laboratory and Clinical Tests

Upon admission, blood samples were collected from the cubital vein of each participant after a minimum 10‐h fasting period. The levels of total cholesterol (TC), TG, FBG, and high‐density lipoprotein cholesterol (HDL‐C) were determined using enzymatic assays performed by an automated biochemical analyzer. The Friedewald formula was used to calculate low‐density lipoprotein cholesterol (LDL‐C) levels. HbA1c levels were assessed using high‐performance liquid chromatography [36]. The modified biplane Simpson method was employed to assess left ventricular ejection fraction (LVEF) at rest [37]. The triglyceride‐glucose (TyG) index was determined using the following formula: Ln (fasting TG [mg/dL] multiplied by FBG [mg/dL] divided by 2) [38], and participants were categorized into groups (proposed cutoff point of 4.5) [39] based on their baseline TyG values. Controlled glycemia was defined as achieving HbA1c levels ≤ 7% [35]. HTN was characterized by a systolic blood pressure (SBP) of 140 mmHg or higher, a diastolic blood pressure (DBP) of 90 mmHg or higher, or the administration of antihypertensive drugs [40].

### 2.2 Assessment of the CAD Characteristics

A coronary angiogram was performed using standard techniques by two skilled interventional cardiologists. They independently assessed the angiographic data obtained from the catheterization laboratory at Golestan Hospital and documented the characteristics of CAD.

The checklists were designed to gather clinical and demographic data related to diabetes and CAD. The data encompassed information on risk factors for CAD, angiographic data, chronic kidney disease (CKD), blood pressure (BP), LVEF, body mass index (BMI), medications, peripheral artery disease (PAD), New York Heart Association (NYHA) functional class, diabetic therapy, glycemic, lipid profile (HDL‐C, TG, TC, LDL‐C), FBG, HbA1c, and TyG index.

### 2.3 Statistical Analysis

The statistical analysis was conducted using SPSS software (version 24). The Kolmogorov–Smirnov test was employed to evaluate the normality of data distribution. The means and standard deviations (SD) of the continuous variables were reported, and an independent *t*‐test was used, while the categorical variables were expressed as percentages and analyzed using the chi‐square test. A multivariable logistic regression analysis was conducted to identify parameters associated with inadequate glycemic control. The findings were reported as odds ratios (OR) along with their corresponding 95% confidence intervals (CI). The independent variables included in the multivariate analysis were those that demonstrated a *p*‐value of less than 0.05 in the univariate analysis. In addition, a two‐tailed *p*‐value of less than 0.05 was deemed to be statistically significant.

## 3 Results

In this study, determinants of poor glycemic control in 700 T2DM patients with CAD were assessed, consisting of 458 males and 242 females. The mean age of the participants was 60.51 ± 9.76 years, with an age range of 36 to 80 years. Approximately 46.6% of the patients were within the age bracket of 57 to 67 years. A summary of the demographic and clinical characteristics of the patients stratified by glycemic control is provided in Table 1. Based on their glycemic control status, the patients were categorized into two groups: Group 1 or the case group with uncontrolled glycemia (HbA1c > 7%, *n* = 350), and Group 2 or controls with controlled glycemia (HbA1c ≤ 7%, *n* = 350). The study revealed that HTN was the most prevalent risk factor, affecting 64.1% of the patients. Dyslipidemia was present in 50.6% of the patients, followed by smoking at 24%.

**Table 1:** Demographic and clinical characteristics of the patients based on glycemic control status.

Demographic and clinical characteristics		Total (*n* = 700)	Group 1 (Cases)	Group 2 (Controls)	*p*‐value
			(HbA1c > 7%)	(HbA1c ≤ 7%)	
			(*n* = 350)	(*n* = 350)	
		**Mean ± SD**			
Age (years)		60.51 ± 9.76	58.14 ± 10.30	62.88 ± 8.57	< 0.001
Duration of diabetes (years)		7.20 ± 4.51	10.41 ± 4.03	4.25 ± 2.02	< 0.001
BMI (kg/m^2^)		27.10 ± 3.70	27.85 ± 3.97	26.35 ± 3.25	< 0.001
		***n* (%)**			
Gender	Male	458 (65.4)	209 (59.7)	249 (71.1)	< 0.01
	Female	242 (34.6)	141 (40.3)	101 (28.9)	
Age group (years)	35–45	43 (6.1)	30 (8.6)	13 (3.7)	< 0.01
	46–56	158 (22.6)	91 (26)	67 (19.1)	
	57–67	326 (46.6)	162 (46.3)	164 (46.9)	
	68–80	173 (24.7)	67 (19.1)	106 (30.3)	
BMI (kg/m^2^)	18.5–24.9	180 (25.7)	67 (19.1)	113 (32.3)	< 0.001
	25–29.9	369 (52.7)	177 (50.6)	192 (54.9)	
	≥ 30	151 (21.6)	106 (30.3)	45 (12.9)	
SBP (mmHg)	< 140	405 (57.9)	207 (59.1)	198 (56.6)	0.540
	≥ 140	295 (42.1)	143 (40.9)	152 (43.4)	
DBP (mmHg)	< 90	305 (43.6)	143 (40.9)	162 (46.3)	0.170
	≥ 90	395 (56.4)	207 (59.1)	188 (53.7)	
Prior revascularization^a^		214 (30.6)	113 (32.3)	101 (28.9)	0.367
Prior MI		154 (22)	105 (30)	49 (14)	< 0.001
Family history of CAD		93 (13.3)	50 (14.3)	43 (12.3)	0.504
Prior stroke		123 (17.6)	68 (19.4)	55 (15.7)	0.233
Dyslipidemia		354 (50.6)	206 (58.9)	148 (42.3)	< 0.001
HTN		449 (64.1)	242 (69.1)	207 (59.1)	< 0.01
PAD		100 (14.3)	69 (19.7)	31 (8.9)	< 0.001
CKD		58 (8.3)	47 (13.4)	11 (3.1)	< 0.001
Current smoking		168 (24)	99 (28.3)	69 (19.7)	< 0.05

Abbreviations: BMI, body mass index; CAD, coronary artery disease; CKD, chronic kidney disease; DBP, diastolic blood pressure; HTN, hypertension; MI, myocardial infarction; PAD, peripheral artery disease; SBP, systolic blood pressure.

^a^ Revascularization included coronary artery bypass grafting and percutaneous coronary intervention.

Notably, patients with uncontrolled glycemia were generally younger, had a higher prevalence of dyslipidemia, smoking, PAD, CKD, and previous MI, longer diabetes duration, and elevated BMI compared to those with optimal glycemic control (*p* < 0.05). There were substantial age and gender differences between the case and control groups (*p* < 0.05). Additionally, 369 patients were overweight, and 151 were obese. The mean BMI significantly differed between the two groups (*p* < 0.001). However, no substantial differences were observed between the groups regarding the prevalence of cardiovascular medications, left main CAD, BP, history of prior stroke, previous revascularization procedures, and family history of CAD (*p* > 0.05).

A summary of cardiovascular and medication parameters of the patients stratified by glycemic control is displayed in Table 2. There were significant differences in the prevalence of NYHA functional class, angiographic findings (including severe calcification, type B2/C lesions, chronic total occlusion (CTO) lesions, thrombotic lesions, and ostial lesions), as well as the use of oral glucose‐lowering medications and insulin between the two groups (*p* < 0.05). Furthermore, 42.6% of patients exhibited triple vessel disease, and 60% of these patients had HbA1c levels > 7%. Notably, patients with uncontrolled glycemia had lower LVEF compared to those with optimal glycemic control (*p* < 0.001).

**Table 2:** Cardiovascular and medication parameters of patients based on glycemic control status (*n* = 700).

Parameters		Total (*n* = 700)	Group 1 (cases)			Group 2 (controls)	*p*‐value
			HbA1c > 7% (*n* = 350)			HbA1c ≤ 7 % (*n* = 350)	
		**Mean ± SD**					
LVEF (%)		58.80 ± 5.03	56.16 ± 5.92			61.44 ± 1.28	< 0.001
		***n* (%)**					
NYHA functional class	I	339 (48.4)	140 (40)			199 (56.9)	< 0.001
	II	259 (37)	136 (38.9)			123 (35.1)	
	III	102 (14.6)	74 (21.1)			28 (8)	
Angiographic data							
Three‐vessel disease		298 (42.6)	178 (50.9)			120 (34.3)	< 0.001
Severe calcification		65 (9.3)	45 (12.9)			20 (5.7)	< 0.01
CTO lesion		90 (12.9)	59 (16.9)			31 (8.9)	< 0.01
Thrombotic lesion		50 (7.1)	37 (10.6)			13 (3.7)	< 0.01
Type B2/C lesion		348 (49.7)	188 (53.7)			160 (45.7)	< 0.05
Ostial lesion		83 (11.9)	56 (16)			27 (7.7)	< 0.01
Left main CAD		88 (12.6)	52 (14.9)			36 (10.3)	0.087
Cardiovascular medications							
Statins		647 (92.4)	323 (92.3)			324 (92.6)	> 0.99
β‐blocker		618 (88.3)	305 (87.1)			313 (89.4)	0.411
ACEI/ARB		196 (28)	102 (29.1)			94 (26.9)	0.556
Aspirin		514 (73.4)	253 (72.3)			261 (74.6)	0.549
Antidiabetic therapy							
Oral glucose‐lowering medications		518 (74)	218 (62.3)			300 (85.7)	< 0.001
Insulin therapy		182 (26)	132 (37.7)			50 (14.3)	< 0.001

Abbreviations: ACEI, angiotensin‐converting enzyme inhibitor; ARB, angiotensin II receptor blocker; CAD, coronary artery disease; CTO, chronic total occlusion; LVEF, left ventricular ejection fraction; NYHA, New York Heart Association; PAD, peripheral artery disease.

Glycemic and lipid profile data of patients based on glycemic control status have been summarized in Table 3. Patients with uncontrolled glycemia had greater levels of HbA1c, FBG, TG, TyG, TC, and LDL‐C than those with optimal glycemic control (*p* < 0.05).

**Table 3:** Glycemic and lipid profile data of patients based on glycemic control status (*n* = 700).

Glycemic and lipid parameters		Total (*n* = 700)	Group 1 (Cases)	Group 2 (Controls)	*p*‐value
			(HbA1c > 7%)	(HbA1c ≤ 7%)	
			(*n* = 350)	(*n* = 350)	
		**Mean ± SD**			
TyG index		4.95 ± 0.79	5.69 ± 0.41	4.22 ± 0.14	< 0.001
HbA1c (%)		7.14 ± 0.91	8.02 ± 0.31	6.26 ± 0.17	< 0.001
FBG (mg/dL)		141.38 ± 26.01	163.31 ± 9.51	119.45 ± 17.33	< 0.001
TC (mg/dL)		164.12 ± 41.10	168.29 ± 42.82	159.71 ± 38.68	< 0.01
TG (mg/dL)		157.94 ± 50.21	167.34 ± 55.42	148.53 ± 45.58	< 0.001
LDL‐C (mg/dL)		90.02 ± 39.06	93.02 ± 40.62	87.01 ± 37.26	< 0.05
HDL‐C (mg/dL)		42.39 ± 12.09	41.80 ± 12.04	42.99 ± 12.21	0.191
		***n* (%)**			
TC (mg/dL)	< 200	576 (82.3)	278 (79.4)	298 (85.1)	< 0.05
	≥ 200	124 (17.7)	72 (20.6)	52 (14.9)	
TG (mg/dL)	< 150	292 (41.7)	12 (3.4)	280 (80)	< 0.001
	≥ 150	408 (58.3)	338 (96.6)	70 (20)	
LDL (mg/dL)	< 100	437 (62.4)	209 (59.7)	228 (65.1)	0.160
	≥ 100	263 (37.6)	141 (40.3)	122 (34.9)	
TyG	< 4.49	346 (49.4)	8 (2.3)	338 (96.6)	< 0.001
	≥ 4.49	354 (50.6)	342 (97.7)	12 (3.4)	
HDL (mg/dL)	< 40	289 (41.3)	145 (41.4)	144 (41.1)	> 0.99
	≥ 40	411 (58.7)	205 (58.6)	206 (58.9)	
FBG (mg/dL)	80–100	54 (7.7)	0 (0)	54 (15.4)	< 0.001
	101–125	264 (37.7)	8 (2.3)	256 (73.1)	
	≥ 126	382 (54.6)	342 (97.7)	40 (11.4)	

Abbreviations: FBG, fasting blood glucose; HbA1c, hemoglobin A1c; HDL‐C, high‐density lipoprotein cholesterol; LDL‐C, low‐density lipoprotein cholesterol; TC, total cholesterol; TG, triglycerides; TYG, triglyceride‐glucose index.

Table 4 shows a summary of the multivariate logistic regression analysis of factors associated with poor glycemic control. Significant independent predictors of inadequate glycemic control were identified as diabetes duration > 5 years (OR = 1.23, 95% CI: 1.12–1.33; *p* < 0.001), BMI ≥ 25 kg/m^2^(OR = 1.18, 95% CI: 1.10–1.29; *p* < 0.001), and higher TyG index (OR = 1.32, 95% CI: 1.21–1.44; *p* < 0.001).

**Table 4:** Multivariate logistic regression analysis of factors associated with poor glycemic control.

Parameters	OR (95% CI)	*p*‐value
Duration of diabetes (> 5 years)	1.23 (1.12–1.33)	< 0.001
Male gender	0.71 (0.46–1.10)	0.128
TyG	1.32 (1.21–1.44)	< 0.001
BMI (≥ 25 kg/m^2^)	1.18 (1.10–1.29)	< 0.001
TG (≥ 150 mg/dL)	0.97 (0.89–1.07)	0.516

Abbreviations: BMI, body mass index; CI, confidence interval; OR, odds ratio; TG, triglycerides; TyG, triglyceride‐glucose index.

## 4 Discussion

In this case‐control study, determinants of poor glycemic control in 700 T2DM patients with CAD (458 males and 242 females) were assessed. Patients were categorized based on their glycemic control status into two groups: those with uncontrolled glycemia (HbA1c > 7%, *n* = 350) and those with controlled glycemia (HbA1c ≤ 7%, *n* = 350). The mean age of the participants was 60.51 ± 9.76 years (ranged from 36 to 80 years). In addition, 46.6% of the patients were between the ages of 57 and 67. It has been reported that CAD was most prevalent in elderly patients [41]. A study conducted in Iran examined the prevalence and patterns of common CAD risk factors over 11 years, considering gender and age variations and their influence on the age of CAD diagnosis [42]. It was found that 31.5% of the participants were female and 68.5% were male. Men were typically diagnosed with CAD at a younger age than women and presented with fewer risk factors at the time of diagnosis [42]. Despite the lower CVD prevalence in women compared to men, women have higher mortality rates and worse prognoses after an acute cardiovascular event [43]. Specifically, females with acute coronary disease often have more unfavorable short‐term and long‐term outcomes compared to males [44]. It has been revealed that women with CAD are less likely to undergo coronary angiography than men and present with more severe clinical symptoms [45, 46, 47]. It was also reported that inadequate glycemic control was more prevalent in women than in men [48, 49]. Multiple hypotheses have been proposed to elucidate this observation, including variations in BMI, glucose metabolism, psychosocial factors, and hormonal differences between men and women [50].

Half of the patients (*n* = 369) in this study were identified as overweight, while 21.6% (*n* = 151) were classified as obese. A retrospective study in Greece among 362 individuals with a history of CAD, predominantly male (88%) with an average age of 67 years, found that 49.2% were overweight and 32.9% were obese [51]. It has been indicated that there is a link between obesity and an increased susceptibility to CAD [52, 53].

In this study, HTN was the most prevalent risk factor, affecting 64.1% of the patients. Dyslipidemia was present in 50.6% of them, followed by smoking at 24%. The findings indicated that patients with uncontrolled glycemia were younger, had a higher prevalence of dyslipidemia, smoking, HTN, PAD, CKD, and previous MI, longer diabetes duration, lower LVEF, elevated BMI, and greater levels of HbA1c, FBG, TG, TyG, TC, and LDL‐C compared to those with optimal glycemic control. Previous studies have emphasized the significant association between HTN and CAD [54, 55, 56]. Furthermore, dyslipidemia, characterized by elevated levels of TG or LDL, is linked to heightened cardiovascular risks [57]. It was reported that a prolonged duration of diabetes is related to inadequate glycemic control [58, 59]. A study reported that patients with diabetes for more than 15 years are at the highest risk of developing HF. It was stated that for every additional 5 years of diabetes duration, there is a 17% relative increase in the likelihood of experiencing HF [60].

Poor glycemic control in diabetic patients is influenced by a variety of predictors, which can be categorized into medication adherence [61, 62], education [61], and lifestyle factors such as smoking and obesity [63]. In this study, diabetes duration > 5 years, BMI ≥ 25 kg/m², and higher TyG index were significant independent predictors of poor glycemic control. It has been reported that a duration of diabetes greater than 5 years is consistently linked to poor glycemic control, with studies showing that longer duration correlates with increased complications and higher HbA1c levels [64, 65]. For instance, one study found that a duration of over 7 years significantly increased the odds of poor glycemic control [64]. A BMI of 25 kg/m² or greater is associated with poor glycemic control, as higher BMI often correlates with insulin resistance [66]. It has been indicated that patients with a BMI above 23 kg/m² are at a higher risk of poor glycemic outcomes [66]. The TyG index, a marker of insulin resistance, shows that values of 4.49 or higher are predictive of poor glycemic control, emphasizing the role of metabolic health in diabetes management [61].

The current study indicated that there were substantial differences in the prevalence of the NYHA functional class, angiographic findings (including severe calcification, type B2/C lesions, CTO lesions, thrombotic lesions, and ostial lesions), and the use of oral glucose‐lowering medications and insulin between Groups 1 and 2. Previous studies have consistently supported a positive association between high FBS and the vulnerability to cardiovascular conditions in diabetic patients [67, 68]. It was reported that among patients with HF and diabetes, a 1% increase in HbA1c levels was linked to a 1.13% higher risk ratio for hospitalization due to cardiac‐related problems and a 1.11% increase in mortality rates [69].

In this present study, 42.6% of the patients exhibited triple vessel disease in this study and 60% of these patients had HbA1c level > 7%. Diabetes is often associated with more complex cases of CAD, characterized by multivessel involvement. Elevated levels of HbA1c are correlated with significant coronary artery involvement [70]. As a result, patients with diabetes typically require surgical revascularization, which contributes to increased healthcare costs [71]. Elevated HbA1c levels were related to an increased likelihood of CVD and mortality [72].

Half of the participants in this study had a TyG index higher than 4.49. Several studies have explored the potential association between the TyG index and various metabolic disorders [73, 74, 75] as well as vascular conditions [76, 77]. The TyG index, a composite measure of fasting TG and glucose levels, has been correlated with the development of coronary artery calcification, a widely recognized surrogate marker for evaluating the risk of CVD [78]. It has been suggested that disturbances in glucose metabolism can expedite the development of atherosclerotic plaques, culminating in plaque rupture, thrombosis, and endothelial dysfunction [79]. Furthermore, an elevated TyG index has been notably related to an increased susceptibility to both cardiovascular and metabolic disorders [80, 81]. One study demonstrated a correlation between elevated FBS levels and the TyG index with an increased susceptibility to cardiac complications [81]. The incorporation of FBS and the TyG index could predict cardiac complications [81].

The quantitative measurement of HbA1c levels is a widely used, reliable, and straightforward method for assessing glycemic control [82, 83]. Patients with diabetes are expected to experience a higher lifetime cardiovascular risk compared with those without diabetes, despite having similar 5‐year risks [84]. Various factors, including age over 40 years, male gender, and the presence of clinical and biochemical indicators such as HTN, elevated LDL levels, and renal dysfunction, can increase the vulnerability to CVDs [84]. It has been indicated that rigorous glycemic control may reduce the incidence of cardiovascular events [85]. Personalized self‐efficacy education can enhance the metabolic profiles of patients with diabetes [86]. In addition, some dietary interventions may improve glycemic control and reduce cardiometabolic risk factors [87, 88, 89, 90, 91, 92].

This study is one of a limited number of studies that were conducted among T2DM patients with CAD in Iran [33, 34]. The results highlight the relationship between cardiovascular health status, glycemic control, and various clinical factors in T2DM patients with CAD. Furthermore, it provided significant insights into the burden of T2DM in patients with CAD and identified clinical parameters that were associated with inadequate glycemic control. The results could improve the understanding of risk factors and disease management levels in a middle‐income or Middle Eastern country. This study had several limitations, notably, it was a single‐center observational study. In addition, levels of physical activity and dietary intake patterns were not assessed. It is recommended that further studies include a larger sample size, extended duration, and multiple centers to improve the quality of outcomes. Additional prospective, interventional, and longitudinal studies are required to investigate the effects of blood glucose control and its enhancement on the clinical outcomes of diabetes patients with CAD.

## 5 Conclusion

This study revealed that CAD patients with good glycemic control had better clinical and cardiovascular status. Patients with uncontrolled glycemia were younger, exhibited longer duration of diabetes, lower LVEF, elevated BMI, and higher levels of lipid profiles compared to those with optimal glycemic control. Furthermore, there were substantial differences in the prevalence of NYHA functional class and angiographic findings between the two groups. Diabetes duration, BMI, and TyG index were significant independent predictors of glycemic control. HbA1c may serve as a valuable parameter for risk stratification in these patients. It highlighted the negative consequences associated with poor glycemic control and emphasized the importance of maintaining lower HbA1c levels in these patients. These insights are crucial for healthcare practitioners in tailoring diabetes management strategies and interventions to reduce the risk of diabetes‐related or CAD‐related complications in T2DM with CAD.

## Author Contributions

**Habib Haybar:** conceptualization, investigation, writing – review and editing, methodology, supervision, project administration. **Maryam Memar:** data curation, methodology, writing – review and editing, conceptualization, investigation. **Seyed Masoud Seyedian:** conceptualization, methodology, writing – review and editing, supervision. **Shirin Azizidoost:** writing – review and editing, conceptualization, methodology. **Shooka Mohammadi:** writing – original draft, writing – review and editing, data curation, formal analysis.

## Funding

The authors received no specific funding for this work.

## Ethics Statement

The Medical Ethics Committee of Ahvaz Jundishapur University of Medical Sciences (AJUMS) granted ethical approval for this study (No. IR. AJUMS. HGOLESTAN. REC.1401.150).

## Conflicts of Interest

The authors declare no conflicts of interest.

## Transparency Statement

The lead author Seyed Masoud Seyedian affirms that this manuscript is an honest, accurate, and transparent account of the study being reported; that no important aspects of the study have been omitted; and that any discrepancies from the study as planned (and, if relevant, registered) have been explained.

## Data Availability

The data that support the findings of this study are available from the corresponding author upon reasonable request.
